# Supplementary material for: The combined influence of chronic kidney disease and peripheral artery disease on long-term all-cause and cardio-cerebrovascular disease mortality among middle-aged and elderly individuals: A nationwide cohort study
Source: PLoS One. 2025 Dec 5;20(12):e0336338. doi: 10.1371/journal.pone.0336338 (PMC12680168; doi:10.1371/journal.pone.0336338)
Supplement: S1 Table — (DOCX) [file pone.0336338.s001.docx]

**Supplementary Table 1.​ Definitions and classification criteria for covariates included in the analysis.**

| **Variable** | **Definition and Classification Criteria** |
| --- | --- |
| ***Family poverty income ratio*** | Income was assessed using the poverty income ratio (PIR, the ratio of family income divided by a poverty threshold specific for family size using guidelines from the US Department of Health and Human Services) and categorized as ≤1.0, 1.1-3.0 and >3.0 [1]. |
| ***Smoking status*** | Never smokers were classified as those who reported smoking <100 cigarettes during their lifetime. Those who smoked >100 cigarettes in their lifetime were considered as current smokers, and those who smoked >100 cigarettes and had quit smoking were considered as former smokers [2]. |
| ***Drinking status*** | Drinking status was classified as nondrinker, low-to-moderate drinker (<2 drinks/day in men and <1 drink/day in women), or heavy drinker (≥2 drinks/day in men and ≥1 drinks/day in women) [2]. |
| ***Physical activity*** | Physical activity was categorized as inactive group (no leisure-time physical activity), insufficiently active group (leisure time moderate activity 1–5 times per week with MET ranging from 3 to 6 or leisure-time vigorous activity 1–3 times per week with MET >6), or active group (those who had more leisure-time moderate-or-vigorous activity than above) [3]. |
| ***Healthy Eating Index*** | The Healthy Eating Index (HEI) is a measure calculated from 24-hour dietary recall data to assess diet quality based on the 2015–2020 Dietary Guidelines for Americans (DGA) [4]. It comprises 13 subgroups, with a total possible score of 100. Nine components evaluate adequacy (higher intakes contribute to a higher score) including total fruits, whole fruits, total vegetables, greens and beans, whole grains, dairy, total protein foods, seafood and plant proteins, and fatty acids. The remaining four components assess moderation (lower intakes yield a higher score), covering refined grains, sodium, added sugars, and saturated fats. The HEI offers a comprehensive framework for understanding dietary patterns and their relationship to health outcomes, with scores reflecting adherence to key dietary recommendations. |
| ***Diabetes*** | The diagnostic criteria for diabetes encompass the following parameters: doctor told you have diabetes; glycohemoglobin HbA1c (%) ≥ 6.5; fasting glucose (mmol/l) ≥ 7.0; random blood glucose (mmol/l) ≥ 11.1; two-hour oral glucose tolerance test (OGTT) blood glucose (mmol/l) ≥ 11.1; use of diabetes medication or insulin [5]. |
| ***Hypertension*** | Average blood pressure was calculated according to the following protocol: The diastolic reading with zero is excluded from diastolic average calculations; in instances where all diastolic readings were zero, the average is considered zero; if only one blood pressure reading was obtained, that singular reading is designated as the average; in cases of multiple blood pressure readings, the initial reading is consistently omitted from the calculated average. Hypertension was defined as a systolic blood pressure of 140 mmHg or higher, a diastolic blood pressure level of 90 mmHg or higher, or the self-reported use of antihypertensive medications, or a medical diagnosis of hypertension by a healthcare professional [6]. |
| ***Hyperlipidemia*** | The Adult Treatment Panel III (ATP III) of the National Cholesterol Education Program (NCEP) defines hyperlipidemia as elevated levels of total cholesterol (≥200 mg/dL), triglycerides (≥150 mg/dL), low-density lipoprotein (LDL) cholesterol (≥130 mg/dL), or low levels of high-density lipoprotein (HDL) cholesterol (<40 mg/dL in males or <50 mg/dL in females) [7]. Additionally, individuals using cholesterol-lowering medications are also classified as having hyperlipidemia. |

**References**

1. **Services USDoHaH. Poverty Guidelines, Research, and Measurement** [<http://aspe.hhs.gov/POVERTY/index.shtml>.]

2. Qiu Z, Chen X, Geng T, Wan Z, Lu Q, Li L, Zhu K, Zhang X, Liu Y, Lin X *et al*: **Associations of Serum Carotenoids With Risk of Cardiovascular Mortality Among Individuals With Type 2 Diabetes: Results From NHANES**. *Diabetes Care* 2022, **45**(6):1453-1461.

3. Beddhu S, Baird BC, Zitterkoph J, Neilson J, Greene T: **Physical activity and mortality in chronic kidney disease (NHANES III)**. *Clin J Am Soc Nephrol* 2009, **4**(12):1901-1906.

4. Kirkpatrick SI, Reedy J, Krebs-Smith SM, Pannucci TE, Subar AF, Wilson MM, Lerman JL, Tooze JA: **Applications of the Healthy Eating Index for Surveillance, Epidemiology, and Intervention Research: Considerations and Caveats**. *J Acad Nutr Diet* 2018, **118**(9):1603-1621.

5. Li W, Peng J, Shang Q, Yang D, Zhao H, Xu H: **Periodontitis and the risk of all-cause and cause-specific mortality among US adults with diabetes: A population-based cohort study**. *J Clin Periodontol* 2023.

6. Tan L, Liu Y, Liu J, Zhang G, Liu Z, Shi R: **Association between insulin resistance and uncontrolled hypertension and arterial stiffness among US adults: a population-based study**. *Cardiovasc Diabetol* 2023, **22**(1):311.

7. **Third Report of the National Cholesterol Education Program (NCEP) Expert Panel on Detection, Evaluation, and Treatment of High Blood Cholesterol in Adults (Adult Treatment Panel III) final report**. *Circulation* 2002, **106**(25):3143-3421.
