# Supplementary material for: The combined influence of chronic kidney disease and peripheral artery disease on long-term all-cause and cardio-cerebrovascular disease mortality among middle-aged and elderly individuals: A nationwide cohort study
Source: PLoS One. 2025 Dec 5;20(12):e0336338. doi: 10.1371/journal.pone.0336338 (PMC12680168; doi:10.1371/journal.pone.0336338)
Supplement: S2 Table — (DOCX) [file pone.0336338.s002.docx]

| **Covariate** |  |  |  |  |  | **VIF** |
| --- | --- | --- | --- | --- | --- | --- |
| Age |  |  |  |  |  | 1.21 |
| Sex |  |  |  |  |  | 1.10 |
| Race/Ethnicity |  |  |  |  |  | 1.32 |
| Education Level |  |  |  |  |  | 1.15 |
| Poverty Income Ratio |  |  |  |  |  | 1.26 |
| Smoking Status |  |  |  |  |  | 1.34 |
| Drinking status |  |  |  |  |  | 1.12 |
| Body Mass Index |  |  |  |  |  | 1.38 |
| Physical activity |  |  |  |  |  | 1.36 |
| Hypertension |  |  |  |  |  | 1.49 |
| Diabetes Mellitus |  |  |  |  |  | 1.41 |
| Hyperlipidemia |  |  |  |  |  | 1.23 |
| Healthy Eating Index (HEI) Score |  |  |  |  |  | 1.18 |
| History of Cardiovascular Disease |  |  |  |  |  | 1.44 |
| History of Cancer |  |  |  |  |  | 1.19 |

**Supplementary Table 2.** Variance inflation factor (VIF) values for covariates included in the multivariable Cox regression model

**Note:** All variance inflation factor (VIF) values were <5, indicating no significant multicollinearity among covariates included in the multivariable Cox regression models.
