# Supplementary material for: The combined influence of chronic kidney disease and peripheral artery disease on long-term all-cause and cardio-cerebrovascular disease mortality among middle-aged and elderly individuals: A nationwide cohort study
Source: PLoS One. 2025 Dec 5;20(12):e0336338. doi: 10.1371/journal.pone.0336338 (PMC12680168; doi:10.1371/journal.pone.0336338)
Supplement: S3 Table — (DOCX) [file pone.0336338.s003.docx]

**Supplementary Table 3.** Baseline characteristics of the middle-aged and older participants by CKD in NHANES 1999–2004.

| **Characteristics** | **Total (n=7243)** | **CKD** | | ***P* value** |
| --- | --- | --- | --- | --- |
|  |  | **No (n=5488)** | **Yes (n=1755)** |  |
| Age, years |  |  |  | <0.001 |
| 40-59 | 3456(65.11) | 3080(72.02) | 376(33.32) |  |
| ≥60 | 3787(34.89) | 2408(27.98) | 1379(66.68) |  |
| Sex, % |  |  |  | <0.001 |
| Female | 3534(50.93) | 2664(50.09) | 870(54.80) |  |
| Male | 3709(49.07) | 2824(49.91) | 885(45.20) |  |
| Race/ethnicity, % |  |  |  | 0.004 |
| Non-Hispanic White | 3966(78.29) | 2952(78.88) | 1014(75.56) |  |
| Non-Hispanic Black | 1253(8.77) | 933(8.36) | 320(10.66) |  |
| Other race | 2024(12.94) | 1603(12.76) | 421(13.78) |  |
| Living status, % |  |  |  | <0.001 |
| With partners | 2458(29.24) | 1689(26.77) | 769(40.60) |  |
| Alone | 4785(70.76) | 3799(73.23) | 986(59.40) |  |
| Education level, % |  |  |  | <0.001 |
| Below high school | 2443(19.86) | 1735(17.68) | 708(29.86) |  |
| High school | 1689(26.05) | 1279(26.01) | 410(26.23) |  |
| Above high school | 3111(54.09) | 2474(56.31) | 637(43.91) |  |
| Family PIR, % |  |  |  | <0.001 |
| ≤1.0 | 1108(10.21) | 805(9.58) | 303(13.09) |  |
| 1.1–3.0 | 3040(33.30) | 2157(30.67) | 883(45.41) |  |
| >3.0 | 3095(56.49) | 2526(59.75) | 569(41.50) |  |
| Smoking status, % |  |  |  | < 0.001 |
| Never smoker | 3354(46.07) | 2541(46.00) | 813(46.41) |  |
| Former smoker | 2487(33.17) | 1807(32.25) | 680(37.42) |  |
| Current smoker | 1402(20.76) | 1140(21.76) | 262(16.17) |  |
| Drinking status, % |  |  |  | <0.001 |
| Nondrinker | 1865(22.51) | 1300(20.37) | 565(32.34) |  |
| Low-to-moderate drinker | 4746(67.33) | 3672(68.65) | 1074(61.25) |  |
| Heavy drinker | 632(10.16) | 516(10.97) | 116(6.42) |  |
| Body mass index, % |  |  |  | 0.045 |
| <25.0 kg/m^2^ | 1971(28.84) | 1487(29.00) | 484(28.15) |  |
| 25.0-29.9 kg/m^2^ | 2836(38.17) | 2189(38.82) | 647(35.23) |  |
| >29.9 kg/m^2^ | 2436(32.98) | 1812(32.19) | 624(36.62) |  |
| Physical activity, % |  |  |  | <0.001 |
| Inactive | 2276(24.16) | 1569(21.56) | 707(36.17) |  |
| Insufficiently active | 3406(54.49) | 2694(56.53) | 712(45.08) |  |
| Active | 1561(21.35) | 1225(21.91) | 336(18.75) |  |
| HEI | 51.14(42.14,60.43) | 50.92(41.97,60.24) | 52.00(43.08,61.16) | 0.035 |
| Hypertension, % |  |  |  | <0.001 |
| No | 3299(52.86) | 2875(58.08) | 424(28.87) |  |
| Yes | 3944(47.14) | 2613(41.92) | 1331(71.13) |  |
| Diabetes mellitus, % |  |  |  | <0.001 |
| No | 5935(86.83) | 4757(90.33) | 1178(70.69) |  |
| Yes | 1308(13.17) | 731(9.67) | 577(29.31) |  |
| Hyperlipidemia, % |  |  |  | 0.014 |
| No | 1444(19.91) | 1123(20.52) | 321(17.07) |  |
| Yes | 5799(80.09) | 4365(79.48) | 1434(82.93) |  |
| PAD, % |  |  |  | <0.001 |
| No | 6682(94.94) | 5215(96.71) | 1467(86.82) |  |
| Yes | 561(5.06) | 273(3.29) | 288(13.18) |  |
| All-cause mortality, % |  |  |  | <0.001 |
| No | 4395(71.11) | 3838(77.83) | 557(40.19) |  |
| Yes | 2848(28.89) | 1650(22.17) | 1198(59.81) |  |
| CCD mortality, % |  |  |  | <0.001 |
| No | 6483(92.56) | 5092(95.06) | 1391(81.07) |  |
| Yes | 760(7.44) | 396(4.94) | 364(18.93) |  |
| Follow-up time, years | 16.92(15.17,18.67) | 17.17(15.67,18.75) | 14.00(7.33,17.58) | <0.001 |

Abbreviations: PIR, poverty income ratio; HEI, Healthy Eating Index; CKD, chronic kidney disease; PAD, peripheral artery disease; CCD, cardio-cerebrovascular disease.

Continuous variables are presented as medians [interquartile ranges]. Categorical variables are presented as numbers (percentages). Sampling weights were applied for calculation of demographic descriptive statistics; N reflect the study sample while percentages reflect the survey-weighted data.
