# Supplementary material for: The combined influence of chronic kidney disease and peripheral artery disease on long-term all-cause and cardio-cerebrovascular disease mortality among middle-aged and elderly individuals: A nationwide cohort study
Source: PLoS One. 2025 Dec 5;20(12):e0336338. doi: 10.1371/journal.pone.0336338 (PMC12680168; doi:10.1371/journal.pone.0336338)
Supplement: S4 Table — (DOCX) [file pone.0336338.s004.docx]

**Supplementary Table 4.** Baseline characteristics of the middle-aged and older participants by PAD in NHANES 1999–2004.

| **Characteristics** | **Total (n=7243)** | **PAD** | | ***P* value** |
| --- | --- | --- | --- | --- |
|  |  | **No (n=6682)** | **Yes (n=561)** |  |
| Age, years |  |  |  | <0.001 |
| 40-59 | 3456(65.11) | 3373(67.17) | 83(26.36) |  |
| ≥60 | 3787(34.89) | 3309(32.83) | 478(73.64) |  |
| Sex, % |  |  |  | 0.066 |
| Female | 3534(50.93) | 3254(50.66) | 280(55.99) |  |
| Male | 3709(49.07) | 3428(49.34) | 281(44.01) |  |
| Race/ethnicity, % |  |  |  | <0.001 |
| Non-Hispanic White | 3966(78.29) | 3648(78.30) | 318(77.99) |  |
| Non-Hispanic Black | 1253(8.77) | 1125(8.47) | 128(14.40) |  |
| Other race | 2024(12.94) | 1909(13.22) | 115(7.61) |  |
| Living status, % |  |  |  | <0.001 |
| With partners | 2458(29.24) | 2201(28.60) | 257(41.24) |  |
| Alone | 4785(70.76) | 4481(71.40) | 304(58.76) |  |
| Education level, % |  |  |  | <0.001 |
| Below high school | 2443(19.86) | 2196(19.17) | 247(32.79) |  |
| High school | 1689(26.05) | 1544(25.84) | 145(29.99) |  |
| Above high school | 3111(54.09) | 2942(54.99) | 169(37.22) |  |
| Family PIR, % |  |  |  | <0.001 |
| ≤1.0 | 1108(10.21) | 990(9.96) | 118(14.84) |  |
| 1.1–3.0 | 3040(33.30) | 2740(32.33) | 300(51.62) |  |
| >3.0 | 3095(56.49) | 2952(57.71) | 143(33.54) |  |
| Smoking status, % |  |  |  | <0.001 |
| Never smoker | 3354(46.07) | 3165(46.76) | 189(33.07) |  |
| Former smoker | 2487(33.17) | 2251(32.78) | 236(40.46) |  |
| Current smoker | 1402(20.76) | 1266(20.46) | 136(26.47) |  |
| Drinking status, % |  |  |  | < 0.001 |
| Nondrinker | 1865(22.51) | 1691(22.08) | 174(30.62) |  |
| Low-to-moderate drinker | 4746(67.33) | 4407(67.77) | 339(59.01) |  |
| Heavy drinker | 632(10.16) | 584(10.15) | 48(10.37) |  |
| Body mass index, % |  |  |  | 0.443 |
| <25.0 kg/m^2^ | 1971(28.84) | 1794(28.77) | 177(30.20) |  |
| 25.0-29.9 kg/m^2^ | 2836(38.17) | 2623(38.35) | 213(34.92) |  |
| >29.9 kg/m^2^ | 2436(32.98) | 2265(32.88) | 171(34.88) |  |
| Physical activity, % |  |  |  | <0.001 |
| Inactive | 2276(24.16) | 2017(23.35) | 259(39.53) |  |
| Insufficiently active | 3406(54.49) | 3204(55.18) | 202(41.62) |  |
| Active | 1561(21.35) | 1461(21.48) | 100(18.85) |  |
| HEI | 51.14(42.14,60.43) | 51.22(42.18,60.55) | 50.18(41.36,58.95) | 0.178 |
| Hypertension, % |  |  |  | <0.001 |
| No | 3299(52.86) | 3170(54.32) | 129(25.49) |  |
| Yes | 3944(47.14) | 3512(45.68) | 432(74.51) |  |
| Diabetes mellitus, % |  |  |  | <0.001 |
| No | 5935(86.83) | 5545(87.54) | 390(73.44) |  |
| Yes | 1308(13.17) | 1137(12.46) | 171(26.56) |  |
| Hyperlipidemia, % |  |  |  | < 0.001 |
| No | 1444(19.91) | 1351(20.24) | 93(13.59) |  |
| Yes | 5799(80.09) | 5331(79.76) | 468(86.41) |  |
| CKD, % |  |  |  | <0.001 |
| No | 5488(82.14) | 5215(83.67) | 273(53.46) |  |
| Yes | 1755(17.86) | 1467(16.33) | 288(46.54) |  |
| All-cause mortality, % |  |  |  | <0.001 |
| No | 4395(71.11) | 4273(73.46) | 122(27.04) |  |
| Yes | 2848(28.89) | 2409(26.54) | 439(72.96) |  |
| CCD mortality, % |  |  |  | <0.001 |
| No | 6483(92.56) | 6060(93.48) | 423(75.29) |  |
| Yes | 760(7.44) | 622(6.52) | 138(24.71) |  |
| Follow-up time, years | 16.92(15.17,18.67) | 16.92(15.33,18.75) | 10.67(5.33,16.17) | <0.001 |

Abbreviations: PIR, poverty income ratio; HEI, Healthy Eating Index; CKD, chronic kidney disease; PAD, peripheral artery disease; CCD, cardio-cerebrovascular disease.

Continuous variables are presented as medians [interquartile ranges]. Categorical variables are presented as numbers (percentages). Sampling weights were applied for calculation of demographic descriptive statistics; N reflect the study sample while percentages reflect the survey-weighted data.
