# Supplementary material for: The combined influence of chronic kidney disease and peripheral artery disease on long-term all-cause and cardio-cerebrovascular disease mortality among middle-aged and elderly individuals: A nationwide cohort study
Source: PLoS One. 2025 Dec 5;20(12):e0336338. doi: 10.1371/journal.pone.0336338 (PMC12680168; doi:10.1371/journal.pone.0336338)
Supplement: S5 Table — (DOCX) [file pone.0336338.s005.docx]

**Supplementary Table 5.** HRs (95% CIs) of mortality according to the combined influence of CKD and PAD after excluding participants who died within two years of follow-up among middle-aged and elderly individuals in NHANES 1999–2004 (n=7,077).

|  | Crude | |  | Model 1 | |  | Model 2 | |
| --- | --- | --- | --- | --- | --- | --- | --- | --- |
|  | HR (95% CI) | *P* value |  | HR (95% CI) | *P* value |  | HR (95% CI) | *P* value |
| **All-cause mortality** | |  |  |  |  |  |  |  |
| No CKD and PAD | 1 [Reference] |  |  | 1 [Reference] |  |  | 1 [Reference] |  |
| CKD alone | 3.59 (3.26-3.95) | <0.001 |  | 2.27 (2.09-2.47) | <0.001 |  | 1.95 (1.79-2.14) | <0.001 |
| PAD alone | 4.21 (3.31-5.35) | <0.001 |  | 2.73 (2.22-3.36) | <0.001 |  | 2.04 (1.64-2.54) | <0.001 |
| CKD and PAD | 9.33 (7.11-12.25) | <0.001 |  | 4.43 (3.52-5.57) | <0.001 |  | 3.05 (2.37-3.92) | <0.001 |
| **Cardio-cerebrovascular Disease Mortality** | | |  |  |  |  |  |  |
| No CKD and PAD | 1 [Reference] |  |  | 1 [Reference] |  |  | 1 [Reference] |  |
| CKD alone | 4.68 (3.94-5.56) | <0.001 |  | 2.85 (2.42-3.36) | <0.001 |  | 2.24 (1.93-2.60) | <0.001 |
| PAD alone | 5.27 (3.60-7.70) | <0.001 |  | 3.25 (2.28-4.64) | <0.001 |  | 2.44 (1.76-3.38) | <0.001 |
| CKD and PAD | 15.27 (10.99-21.22) | <0.001 |  | 6.82 (5.07-9.19) | <0.001 |  | 4.39 (3.12-6.18) | <0.001 |

Model 1 was adjusted for age (40-59, or ≥60), sex (male or female), and race/ethnicity (Non-Hispanic White, Non-Hispanic Black or Other); Model 2 was adjusted as model 1 plus living status (with partners, or alone), education level (below high school, high school, or above high school), family PIR (≤1.0, 1.1–3.0, or >3.0), smoking status (never smoker, former smoker, or current smoker), drinking status (nondrinker, low-to-moderate drinker, or heavy drinker), BMI (<25.0, 25.0-29.9, or >29.9), physical activity (inactive, insufficiently active, or active), HEI (in quartiles), hypertension (yes or no), diabetes mellitus (yes or no), and hyperlipidemia (yes or no).
