# Supplementary material for: The combined influence of chronic kidney disease and peripheral artery disease on long-term all-cause and cardio-cerebrovascular disease mortality among middle-aged and elderly individuals: A nationwide cohort study
Source: PLoS One. 2025 Dec 5;20(12):e0336338. doi: 10.1371/journal.pone.0336338 (PMC12680168; doi:10.1371/journal.pone.0336338)
Supplement: S6 Table — (DOCX) [file pone.0336338.s006.docx]

**Supplementary Table 6.** HRs (95% CIs) of mortality according to the combined influence of CKD and PAD after excluding participants who had CVD history at baseline among middle-aged and elderly individuals in NHANES 1999–2004 (n=6,109).

|  | Crude | |  | Model 1 | |  | Model 2 | |
| --- | --- | --- | --- | --- | --- | --- | --- | --- |
|  | HR (95% CI) | *P* value |  | HR (95% CI) | *P* value |  | HR (95% CI) | *P* value |
| **All-cause mortality** | |  |  |  |  |  |  |  |
| No CKD and PAD | 1 [Reference] |  |  | 1 [Reference] |  |  | 1 [Reference] |  |
| CKD alone | 3.54 (3.17-3.95) | <0.001 |  | 2.33 (2.12-2.56) | <0.001 |  | 2.01 (1.80-2.25) | <0.001 |
| PAD alone | 4.05 (3.05-5.38) | <0.001 |  | 2.66 (2.07-3.41) | <0.001 |  | 2.05 (1.58-2.66) | <0.001 |
| CKD and PAD | 8.87 (6.30-12.50) | <0.001 |  | 4.23 (3.17-5.64) | <0.001 |  | 2.83 (2.07-3.87) | <0.001 |
| **Cardio-cerebrovascular Disease Mortality** | | |  |  |  |  |  |  |
| No CKD and PAD | 1 [Reference] |  |  | 1 [Reference] |  |  | 1 [Reference] |  |
| CKD alone | 4.91 (3.87-6.24) | <0.001 |  | 3.16 (2.51-3.97) | <0.001 |  | 2.53 (1.99-3.21) | <0.001 |
| PAD alone | 5.04 (3.26-7.79) | <0.001 |  | 3.16 (2.09-4.78) | <0.001 |  | 2.47 (1.68-3.62) | <0.001 |
| CKD and PAD | 15.65 (9.79-25.01) | <0.001 |  | 7.09 (4.70-10.68) | <0.001 |  | 4.59 (2.90-7.27) | <0.001 |

Model 1 was adjusted for age (40-59, or ≥60), sex (male or female), and race/ethnicity (Non-Hispanic White, Non-Hispanic Black or Other); Model 2 was adjusted as model 1 plus living status (with partners, or alone), education level (below high school, high school, or above high school), family PIR (≤1.0, 1.1–3.0, or >3.0), smoking status (never smoker, former smoker, or current smoker), drinking status (nondrinker, low-to-moderate drinker, or heavy drinker), BMI (<25.0, 25.0-29.9, or >29.9), physical activity (inactive, insufficiently active, or active), HEI (in quartiles), hypertension (yes or no), diabetes mellitus (yes or no), and hyperlipidemia (yes or no).
