# Supplementary material for: The combined influence of chronic kidney disease and peripheral artery disease on long-term all-cause and cardio-cerebrovascular disease mortality among middle-aged and elderly individuals: A nationwide cohort study
Source: PLoS One. 2025 Dec 5;20(12):e0336338. doi: 10.1371/journal.pone.0336338 (PMC12680168; doi:10.1371/journal.pone.0336338)
Supplement: S7 Table — (DOCX) [file pone.0336338.s007.docx]

**Supplementary Table 7.** HRs (95% CIs) of mortality according to the combined influence of CKD and PAD after excluding participants who had cancer history at baseline among middle-aged and elderly individuals in NHANES 1999–2004 (n=6,382).

|  | Crude | |  | Model 1 | |  | Model 2 | |
| --- | --- | --- | --- | --- | --- | --- | --- | --- |
|  | HR (95% CI) | *P* value |  | HR (95% CI) | *P* value |  | HR (95% CI) | *P* value |
| **All-cause mortality** | |  |  |  |  |  |  |  |
| No CKD and PAD | 1 [Reference] |  |  | 1 [Reference] |  |  | 1 [Reference] |  |
| CKD alone | 3.75 (3.37-4.18) | <0.001 |  | 2.43 (2.21-2.68) | <0.001 |  | 2.05 (1.84-2.28) | <0.001 |
| PAD alone | 4.26 (3.38-5.36) | <0.001 |  | 2.71 (2.18-3.38) | <0.001 |  | 1.92 (1.52-2.43) | <0.001 |
| CKD and PAD | 10.13 (7.72-13.30) | <0.001 |  | 4.73 (3.74-5.99) | <0.001 |  | 3.02 (2.31-3.94) | <0.001 |
| **Cardio-cerebrovascular Disease Mortality** | | |  |  |  |  |  |  |
| No CKD and PAD | 1 [Reference] |  |  | 1 [Reference] |  |  | 1 [Reference] |  |
| CKD alone | 5.20 (4.29-6.30) | <0.001 |  | 3.27 (2.72-3.94) | <0.001 |  | 2.57 (2.15-3.07) | <0.001 |
| PAD alone | 5.20 (3.66-7.38) | <0.001 |  | 3.17 (2.24-4.49) | <0.001 |  | 2.23 (1.62-3.06) | <0.001 |
| CKD and PAD | 18.39 (13.45-25.14) | <0.001 |  | 8.17 (6.09-10.97) | <0.001 |  | 4.85 (3.42-6.88) | <0.001 |

Model 1 was adjusted for age (40-59, or ≥60), sex (male or female), and race/ethnicity (Non-Hispanic White, Non-Hispanic Black or Other); Model 2 was adjusted as model 1 plus living status (with partners, or alone), education level (below high school, high school, or above high school), family PIR (≤1.0, 1.1–3.0, or >3.0), smoking status (never smoker, former smoker, or current smoker), drinking status (nondrinker, low-to-moderate drinker, or heavy drinker), BMI (<25.0, 25.0-29.9, or >29.9), physical activity (inactive, insufficiently active, or active), HEI (in quartiles), hypertension (yes or no), diabetes mellitus (yes or no), and hyperlipidemia (yes or no).
