# Supplementary material for: The combined influence of chronic kidney disease and peripheral artery disease on long-term all-cause and cardio-cerebrovascular disease mortality among middle-aged and elderly individuals: A nationwide cohort study
Source: PLoS One. 2025 Dec 5;20(12):e0336338. doi: 10.1371/journal.pone.0336338 (PMC12680168; doi:10.1371/journal.pone.0336338)
Supplement: Attachment — Submitted filename: Response to Reviewers.docx [file pone.0336338.s009.docx]

**Dear Editor and Reviewers,**

We would like to express our sincere appreciation for your constructive and insightful comments on our manuscript. We have carefully considered each suggestion and revised the manuscript accordingly to improve its clarity and scientific rigor. In accordance with the journal’s submission requirements, we have submitted the revised manuscript in its final, clean format, without any highlighted or tracked changes. Below, we provide a detailed point-by-point response to each of the reviewers’ comments.

**Reviewer #1:**

**Comment 1:** Study Design and Novelty

The use of a large, nationally representative dataset (NHANES 1999–2004) and the long follow-up duration (median 16.92 years) are important strengths. The study uses robust definitions for CKD and PAD, aligned with KDIGO and established ABI thresholds is the strength of this study. However, the novelty is somewhat limited, as several prior studies have investigated the association between CKD, PAD, and mortality. The manuscript should better articulate how it adds to existing literature (e.g., larger sample, dual markers for CKD, longer follow-up) in the Introduction and Discussion.

Recommendation: Emphasize clearly in the introduction how this study improves upon past work (e.g., simultaneous use of UACR and eGFR, PAD diagnosed by ABI, more complete adjustment for confounders).

**Response 1:** Thank you for this constructive suggestion. We have revised the Introduction to explicitly articulate our study’s incremental contribution, emphasizing the simultaneous KDIGO-aligned definition of CKD using both eGFR and UACR, the ABI-based ascertainment of PAD, the large, nationally representative NHANES 1999–2004 cohort with mortality follow-up through 2019 (median 16.9 years), and the incorporation of complex-survey design (weights, strata, clusters) with comprehensive confounder adjustment. We also added a concise statement on how these features extend prior work that used smaller samples, a single kidney metric, and shorter follow-up.

**Comment 2:** Statistical Methods and Interpretation

- The authors adjusted for a large number of variables in Model 2. Multicollinearity risk is acknowledged, but no specific VIF values are provided.

- Interaction effects between CKD and PAD were not significant (p-int = 0.178 and 0.354), suggesting an additive rather than synergistic effect.

Recommendation:

- Provide specific VIF values to support the claim of no multicollinearity.

- Discuss the lack of statistical interaction more explicitly in the discussion. The current language suggests synergy, which may be overstated.

**Response 2:** Thank you for the helpful suggestions. We have revised the manuscript accordingly: (i) we now report variance inflation factors (VIFs) for all covariates in the fully adjusted model, all <5, to document the absence of concerning multicollinearity (added to Methods/Statistical Analysis and noted in Results; summarized in Table Sx); and (ii) we revised the Discussion to explicitly state the lack of statistical interaction between CKD and PAD and to interpret the joint association as an additive effect rather than synergy.

**Comment 3:** Clarity and Language:

While the manuscript is largely understandable, there are several grammatical issues (e.g., verb agreement, awkward phrasing) and overly lengthy sentences. A language edit by a native English speaker or professional editing service is strongly recommended.

**Response 3:** Thank you for this valuable suggestion. We have conducted a comprehensive language revision to improve clarity and readability, including correction of grammar and verb agreement, removal of awkward phrasing, and shortening of overly long sentences.

**Comment 4:** Discussion of Mechanisms:

The discussion is comprehensive, but the pathophysiological mechanisms linking CKD and PAD are somewhat speculative. While references are cited, the causality remains unclear.

**Response 4:** Thank you for this important point. We have revised the Discussion and explicitly added to the Limitations section that causal inference is a key limitation of our study, given its observational design and single baseline assessments. We also tempered mechanistic language and present potential pathways as hypothesis-generating only.

**Comment 5:** Limitations:

The study mentions some limitations (e.g., cross-sectional CKD/PAD diagnosis, lack of angiography), but should also note unmeasured confounding (e.g., medication use, CKD/PAD treatment adherence) more clearly.

**Response 5:** Thank you for the suggestion. We have revised the Limitations to explicitly acknowledge residual and unmeasured confounding, including limited information on medication use (antiplatelet/anticoagulant therapy, statins, or RAS inhibitor), treatment intensity, and adherence to CKD/PAD management and lifestyle interventions.

**Reviewer #2:**

1. Many citations are missing including sections of the assessment (i.e. how mortality is assessed through NHANES, how blood pressure measurements are taken etc.) They are also missing from the covariates.

**Response 1:** Thank you for highlighting this. We have added explicit citations in the Methods for mortality ascertainment via the NCHS Linked Mortality files, NHANES measurement protocols (blood pressure procedures), and all covariates. In the Supplementary Materials, we now provide variable-level definitions, measurement protocols, and corresponding references for each covariate. We also revised the Limitations to state clearly that, despite extensive adjustment, residual and unmeasured confounding may remain.

**Comment 2:** The exclusion criteria are listed but could be more clearly organized. A table or bullet list summarizing inclusion/exclusion might help.

**Response 2:** Thank you for this helpful suggestion. In response, we have revised the Methods section to present the inclusion and exclusion criteria in a clearly structured, numbered format, and provided corresponding sample counts to enhance clarity. We also aligned this text with Figure 1, which outlines the participant selection process.

**Comment 3:** The rationale behind investigating the combined effects of CKD and PAD should be clearly stated in the introduction or early methods. What is the biological or epidemiologic basis for expecting a synergistic effect?

**Response 3:** Thank you for this insightful comment. We have revised the Introduction to briefly clarify the biological and epidemiological rationale for examining the joint effects of CKD and PAD. Specifically, we now state that, given their shared risk factors and overlapping pathophysiologic pathways, it is clinically relevant to assess whether the coexistence of CKD and PAD confers a greater mortality risk than either condition alone.

**Comment 4:** Clarify why specific covariates were included. For instance, explain how HEI, PIR, or living status relate to mortality outcomes or disease interactions.

**Response 4:** Thank you for this helpful comment. We have revised the Methods section to clarify that covariates were selected a priori based on prior literature linking them to both CKD/PAD and mortality outcomes. We also briefly explained the rationale for including variables such as PIR, HEI, and living status, highlighting their relevance to socioeconomic status, dietary quality, and social support—all of which may influence disease progression and mortality.

**Comment 5:** Provide more details on how participants were excluded, and whether results were re-weighted accordingly. Were analyses stratified by any variables?

**Response 5:** Thank you for this important point. We have clarified the participant exclusion process in the Methods and Figure 1, including reasons and sample sizes at each step. All analyses incorporated NHANES examination weights, and we confirmed that reweighting was not necessary, as the exclusions were consistent with analytic guidelines and did not violate survey design assumptions. We did not perform stratified analyses, as our primary aim was to estimate the independent and joint associations of CKD and PAD with mortality in the full population, while adjusting for key covariates. Stratification was avoided to preserve statistical power, particularly for participants with both conditions. However, we explored potential effect modification through interaction terms, which were not statistically significant.

**Comment 6:** Explain multiple models used (Model 1, Model 2, etc.) These are mentioned without specifying what variables were adjusted in each. Add a note or table to define what’s included in each model.

**Response 6:** Thank you for this helpful suggestion. We have revised the table footnotes to clearly define the variables adjusted for in each model. Model 1 includes age, sex, and race/ethnicity, while Model 2 includes all covariates described in the Methods (demographics, lifestyle, socioeconomic status, and clinical factors). These clarifications are now included in the revised manuscript.

**Comment 7:** Check assumptions of Cox regression more thoroughly. Although Schoenfeld residuals and VIFs are mentioned, results are not reported. Include at least a summary or figure confirming proportional hazard assumption and no multicollinearity.

**Response 7:** Thank you for this important observation. We have revised the Methods section to report the results of diagnostic checks. We now state that the proportional hazards assumption was tested using Schoenfeld residuals and showed no significant violations. We also report that all variance inflation factors (VIFs) in the fully adjusted model were <5, indicating no concerning multicollinearity.

**Comment 8:** Revise overly dense paragraphs. Some sections (e.g. lines 243–313) are overly dense and could be split for better readability. Use subheadings (e.g. Mechanisms, Prior Literature, Clinical Implications) in Discussion.

**Response 8:** Thank you for this helpful recommendation. In response, we revised the Discussion section to improve readability by breaking down overly dense paragraphs into shorter, thematically organized sections to guide the reader and enhance clarity. These changes are reflected in the revised manuscript.

**Comment 9:** Tighten grammar and phrasing

"Patients with PAD was also..." → “Patients with PAD were also...”

“This coexistence is associated with higher rates...” → “The coexistence is associated with...”

Watch subject–verb agreement and plural forms.

**Response 9:** Thank you for pointing this out. We carefully reviewed the manuscript for grammatical issues, including subject–verb agreement, plural forms, and awkward phrasing. Revisions were made throughout the text to improve clarity and accuracy. All changes have been incorporated into the revised manuscript.

**Comment 10:** Avoid vague phrases like “substantially heightened”. Use more precise quantitative language whenever possible, especially in scientific writing. Replace with actual HRs or % differences where appropriate.

**Response 10:** Thank you for this valuable suggestion. We have revised the manuscript to replace vague phrases such as “substantially heightened” with more precise quantitative language. Wherever possible, we now report specific hazard ratios (HRs), percentage differences, or effect estimates to clearly convey the magnitude of associations. These revisions have been incorporated throughout the Results and Discussion sections.

**Comment 11:** Improve clarity in mortality definitions. Distinguish clearly between "all-cause mortality" and "cardio-cerebrovascular mortality" at each mention, especially in the abstract, results, and conclusion.

**Response 11:** Thank you for this helpful comment. We have revised the manuscript to ensure consistent and clear distinction between all-cause mortality and cardio-cerebrovascular mortality throughout the abstract, results, and conclusion.

**Comment 12:** The discussion section repeats the same hazard ratios reported earlier. Instead, focus more on interpretation, implications, and mechanisms than reiterating the same numbers.

**Response 12:** Thank you for the valuable suggestion. We have revised the Discussion section to reduce redundancy and added brief statements highlighting the interpretation of our findings and their clinical implications, focusing on the importance of early identification and integrated management of patients with coexisting CKD and PAD.

**Comment 13:** Acknowledge potential residual confounding. Even after adjustment, acknowledge that unmeasured or residual confounding (e.g., medication use, healthcare access, inflammation markers) may influence results.

**Response 13:** Thank you for this important observation. As suggested, we have revised the Discussion to explicitly acknowledge the possibility of residual and unmeasured confounding, including factors such as medication use, healthcare access, and inflammatory markers, which were not available in our dataset. This limitation has been added to the Limitations subsection of the Discussion.

We hope these revisions meet the expectations of the reviewers and the editorial team. Thank you again for your time and consideration.

Sincerely,

Xuan Li, MD

Corresponding Author

On behalf of all co-authors

Department of Vascular Surgery, Zhejiang Hospital, No. 12 Lingyin Road, Hangzhou, Zhejiang, 310013, China.

E-mail: [lixuandr79@outlook.com](mailto:lixuandr79@outlook.com)
