# Supplementary material for: The combined influence of chronic kidney disease and peripheral artery disease on long-term all-cause and cardio-cerebrovascular disease mortality among middle-aged and elderly individuals: A nationwide cohort study
Source: PLoS One. 2025 Dec 5;20(12):e0336338. doi: 10.1371/journal.pone.0336338 (PMC12680168; doi:10.1371/journal.pone.0336338)
Supplement: Attachment — Submitted filename: Response_to_Reviewers_auresp_2.docx [file pone.0336338.s010.docx]

**Dear Editor and Reviewers,**

We would like to express our sincere appreciation for your constructive and insightful comments on our manuscript. We have carefully considered each suggestion and revised the manuscript accordingly to improve its clarity and scientific rigor. In accordance with the journal’s submission requirements, we have submitted the revised manuscript in its final, clean format, without any highlighted or tracked changes. Below, we provide a detailed point-by-point response to each of the reviewers’ comments.

**Reviewer #1:**

**Comment 1:** Novelty and Contribution

- The introduction now better highlights how this study extends prior work (dual CKD measures, ABI-defined PAD, long follow-up, and complex survey design).

- Suggestion: The authors may further emphasize the public health implications—i.e., how screening for both conditions together may identify a particularly high-risk population that merits more aggressive preventive strategies.

**Response 1:** We appreciate your insightful suggestion. In response, we have revised the Introduction to further highlight the public health implications of our findings. We now emphasize how simultaneous screening for CKD and PAD using simple, widely available measures (eGFR, UACR, and ABI) may help identify a particularly high-risk subgroup that could benefit from targeted preventive interventions and multidisciplinary care.

**Comment 2:** Statistical Analysis and Interpretation

- The inclusion of VIF values (<5) and reporting of Schoenfeld residual checks are appropriate.

- However, the authors should present the VIF results (perhaps in supplementary materials) rather than only stating them in text, for transparency.

- The conclusion now states the joint effect is “additive rather than synergistic,” which is more accurate. This is an important clarification, but the clinical framing (“double jeopardy”) still implies synergy—consider softening this phrasing further.

**Response 2:** We sincerely thank the reviewer for these constructive comments. In accordance with the suggestions, we have added a new supplementary table (Supplementary Table S1) presenting the variance inflation factor (VIF) values for all covariates included in the multivariable Cox regression models. All VIF values were below 5, confirming the absence of significant multicollinearity and enhancing the transparency of our statistical analyses.

Additionally, we have revised the conclusion to soften the clinical phrasing and to emphasize the additive—rather than synergistic—nature of the observed joint effect. These revisions improve both the precision and clarity of the manuscript.

**Comment 3:** Mechanistic Discussion

- The mechanistic explanations are thorough, but still somewhat speculative. While the authors now clearly flag this as hypothesis-generating, they may consider shortening the mechanistic section to avoid overinterpretation and emphasize the epidemiological message of the findings.

**Response 3:** We thank the reviewer for this thoughtful recommendation. In response, we have shortened the mechanistic discussion to avoid overinterpretation and ensure alignment with the observational nature of our study. These revisions help improve the focus and clarity of the discussion.

**Comment 4:** Limitations

- The limitations are more complete, including unmeasured confounding (medication use, inflammatory markers, adherence).

- Suggestion: Add one sentence noting that ABI alone may misclassify PAD in populations with arterial stiffness (common in diabetes and CKD). This nuance would further strengthen the discussion.

**Response 4:** We thank the reviewer for this valuable suggestion. In response, we have revised the Limitations section to acknowledge that the use of ABI alone may lead to misclassification of PAD in individuals with arterial stiffness, particularly among those with diabetes or CKD. This addition provides a more nuanced interpretation and strengthens the discussion of study limitations.

**Comment 5:** Terminology

- Ensure consistent use of “cardio-cerebrovascular disease (CCD)” throughout the text. At times, “CVD” or “cardiovascular” appear instead, which may confuse readers.

**Response 5:** We thank the reviewer for this helpful observation. We have carefully reviewed the entire manuscript and revised the terminology to ensure consistent use of “cardio‑cerebrovascular disease (CCD)” throughout the text, tables, and figure legends to maintain clarity and coherence.

**Comment 6:** References

- The reference list is up to date and comprehensive. However, some highly relevant systematic reviews on CKD–PAD overlap (e.g., meta-analyses of vascular risk in CKD populations) could be cited to further contextualize findings.

**Response 6:** We appreciate the reviewer’s thoughtful suggestion. In response, we have incorporated additional citations to recent systematic reviews and meta‑analyses addressing the overlap between CKD and PAD and the associated vascular risk. These references have been added to the Introduction and Discussion section to provide broader context and strengthen the interpretation of our findings.

We hope these revisions meet the expectations of the reviewers and the editorial team. Thank you again for your time and consideration.

Sincerely,

Xuan Li, MD

Corresponding Author

On behalf of all co-authors

Department of Vascular Surgery, Zhejiang Hospital, No. 12 Lingyin Road, Hangzhou, Zhejiang, 310013, China.

E-mail: [lixuandr79@outlook.com](mailto:lixuandr79@outlook.com)
